# Surgical Techniques for Correction of Penile Paraffinoma

**DOI:** 10.21315/mjms2019.26.6.14

**Published:** 2019-12-30

**Authors:** Syahril Anuar Salauddin, Hamid Ghazali

**Affiliations:** Department of Urology, Hospital Tengku Ampuan Afzan, Pahang, Malaysia

**Keywords:** paraffin, granuloma, silicone, penile augmentation, scrotal flap

## Abstract

**Background:**

Penile augmentation using injection of a foreign body into penile skin was mainly performed by non-medical personnel. Majority of these patients end up with complication of an abnormal mass formation known as penile paraffinoma.

**Methods:**

We described three different surgical techniques for correction of penile paraffinoma based on our single-centre experience. Informed consents were obtained from patients whose photographs were taken during the operation step.

**Results:**

In general, three patients had simple excision biopsy with primary suturing, four patients underwent single stage excision of circumferential granuloma with bilateral scrotal skin flap reconstruction and one patient experienced dual stage procedure. Three of them were injected with paraffin, one with silicone and the remaining four were unable to identify the substance used. All patients successfully underwent the surgical procedure and four of them had minor post-operative surgical site infection and wound gapping.

**Conclusion:**

All patients recovered well and the mean International Index of Erectile Function (IIEF-5) score obtained was 24.25. In our experience, excision biopsy was adequate for focal mass and reconstructive surgery using bilateral scrotal flap was suitable for circumferential mass.

## Introduction

The term paraffinoma was introduced by Newcomer and Grahamin in 1971 to describe abnormal histopathologic findings after the injection of a foreign material (1). Several other terminologies are less commonly found in the literature, such as sclerosing lipogranuloma, penile granuloma and siliconoma. This complication occurs as a result of atypical tissue proliferation and inflammatory process caused by the interaction of a foreign substance with human tissue. The technique of penile augmentation is a practice mainly performed by a non-medical personnel and includes injection of this substance. The patient or his partner who underwent the procedure may experience undesirable symptoms and should seek medical attention.

Non-surgical therapy, such as topical cream or steroid injection, was found to be ineffective. The only successful treatment consisted of performing penile reconstructive surgery. Over the years, multiple surgical techniques have been developed for restoring near-normal penile shape. Here, we described three different surgical techniques for the correction of penile paraffinoma based on our single-centre experience.

## Materials and Methods

The study was a retrospective review of three different surgical techniques performed in eight patients from April 2016 to May 2017. Information reviewed were demographic data, patient presentation, details about the surgical procedure and post-operative outcomes. Written consents were prospectively obtained from patients who had their photographs taken.

## Results

The majority of the patients were Malay (five patients), followed by Chinese (two patients) and Indian (one patient). All patients had their injection a few years before seeking treatment. The mean age of patients was 35.5 years. Four of the patients did not recognise the substance used in the injections; however, three informed the use of paraffin and one of silicone. They presented with complications from the first to eighth year after injection and the most common symptom was pain, either to themselves or to the partner during sexual intercourse. On examination, three patients presented a focal mass formation at the ventral aspect of the penis, whereas five exhibited a circumferential hard mass around the penile shaft extending to the base of the scrotum and suprapubic region (Table 1).

There were three types of surgical techniques:

- Simple excision biopsy and primary suturing
- Single-stage excision of circumferential granuloma with bilateral scrotal skin flap reconstruction
- Dual-stage excision of circumferential granuloma and bilateral scrotal skin flap reconstruction

### Simple Excision Biopsy and Primary Suturing

Three patients were treated using this procedure. All of them presented granuloma formation at the ventral side of the penile shaft. The mass was excised along with the overlying skin, and the lateral edges were sutured together. Interrupted absorbable sutures were used to approximate both edges. Eventually, they had a linear longitudinal scar that did not cause any shortening of the penis. The mean duration of surgery was 125 min (100 min–140 min). None of these patients manifested any complications post-operatively. Erection performance was considered satisfactory compared to that before surgery and the mean score was 24 according to the International Index of Erectile Function (IIEF-5).

### Single Stage Excision of Circumferential Granuloma with Bilateral Scrotal Skin Flap Reconstruction

**Figure 1:**
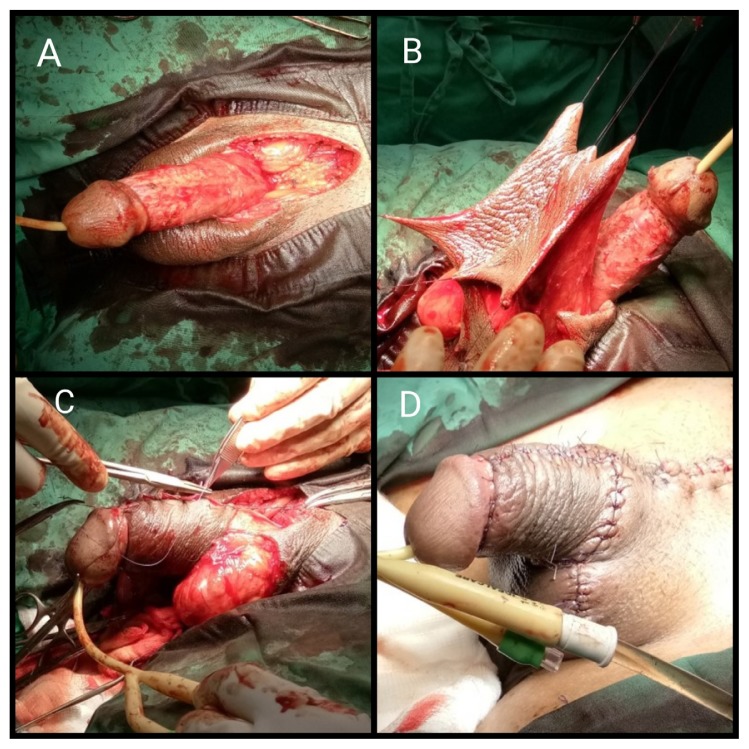
Single stage reconstructive procedure

**Figure 2:**
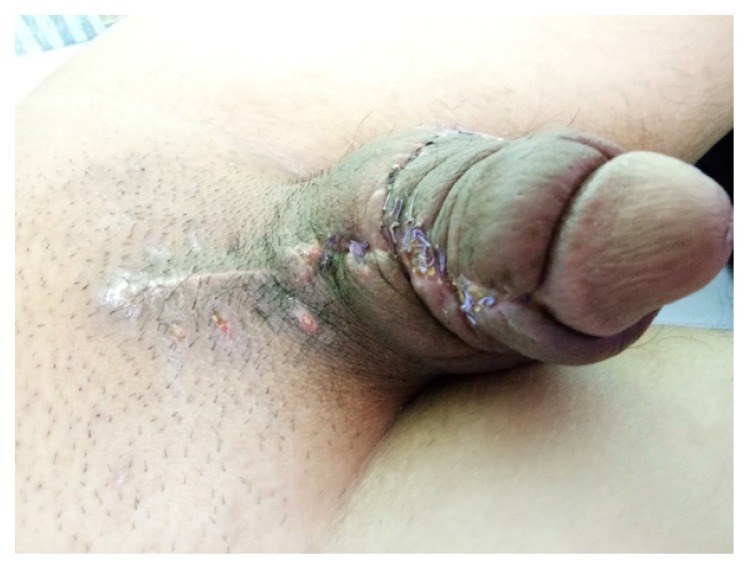
Two weeks after single stage reconstructive procedure

The majority of patients underwent this approach (four patients). All patients presented circumferential penile shaft mass extended until the base of the scrotum and suprapubic region, with one of them also presenting a complication of chronic ulcer over the mass. The steps of surgery were initiated with meticulous excision of the circumferential granuloma on the penile shaft, leaving the superficial fascia covering corpus cavernosum and corpus spongiosum (Figure 1A). Tissue was dissected from the base of the penis and suprapubic region until the whole granuloma tissue was completely excised. The next step consisted of preparing the bilateral scrotal flap by dissecting a thick square shape from the scrotal skin and wrapping over the whole penile shaft (Figure 1B). Accurate measurement was needed depending on the circumference girth of the penile shaft to obtain adequate covering. The graft was approximated using absorbable interrupted sutures, and the remaining wound on the scrotum and suprapubic region were closed (Figures 1C and 1D). The mean duration of the operation was 271 min (225 min–345 min). In this small series of patients, three had minor superficial gapping of the wound edge, which completely healed without secondary intervention. Two weeks post-operatively, the surgical wound was almost recovered, except neo-penile skin oedema that took 6 weeks–8 weeks to fully subside (Figure 2). All patients were satisfied with the outcome and the mean IIEF-5 score post-operatively was 24.5.

### Dual Stage Excision of Circumferential Granuloma and Bilateral Scrotal Skin Flap Reconstruction

**Figure 3:**
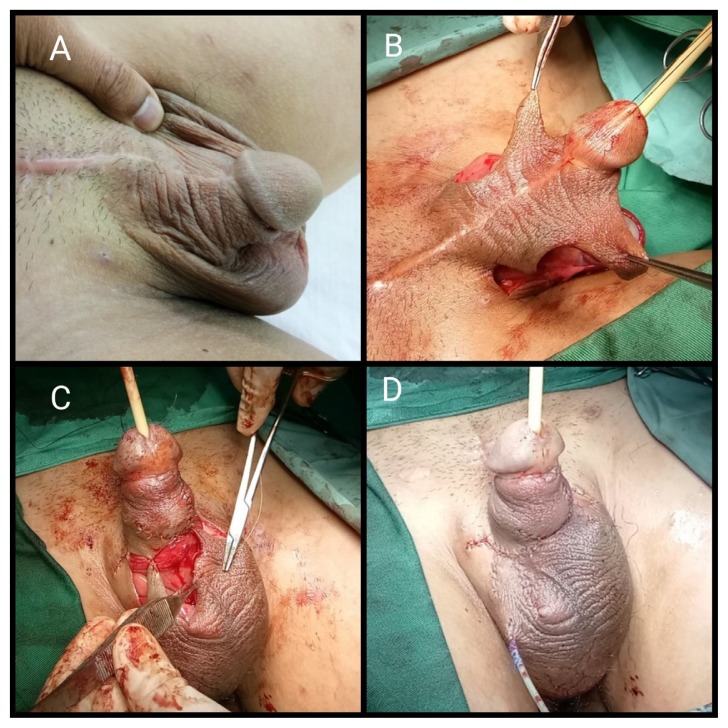
Dual stage reconstructive procedure

Only one patient within this period of observation underwent a dual-stage procedure. The procedure was similar to single-stage excision of circumferential granuloma with bilateral scrotal skin flap reconstruction but was performed on two separate occasions. This patient requested that the procedure be divided into two occasions because he refused general anaesthesia. Therefore, both procedures were performed under combined spinal-epidural anaesthesia. At the first-stage, the granuloma was completely excised followed by burying the penile shaft underneath scrotum skin. A 3-month duration was given for proper healing (Figure 3A). The second stage was the reconstructive procedure. A zigzag incision was made at both lateral aspects of the buried penile shaft. The flap that adhered to the dorsal penile shaft was raised, and the ventral aspect of the penile shaft was excised from the scrotum (Figure 3B). The zigzag flap was opposed ventrally using interrupted suture (Figures 3C and 3D). The first stage took 120 min and the second stage required 260 min. The patient had minor superficial wound dehiscence but recovered completely within 2 weeks and was able to engage in sexual intercourse comfortably, presenting post-operatively with a mean score of 23 according to IIEF-5.

## Discussion

The first person documented to inject a foreign substance into the human body was Robert Gersuny, an Austrian surgeon in 1899. He injected mineral oil (Vaseline) to substitute an absent testis (2) and the immediate success led other medical personnel to test with other materials, such as paraffin and silicone, as part of treatment, especially in the field of plastic surgery. These materials were injected in a semiliquid stage, achieved by heating, before solidification when it gets cooler. Ideally, it must remain stable in the human body and avoid inducing reaction towards the surrounding tissue. Unfortunately, most patients developed complications such as infection, ulceration, contracture, and malignant transformation.

Penile paraffinoma was a common complication after the injection of these materials under the penile skin. In our series, the mean age of patients at the time of injection was 35.5 years, which was similar to that of 32 years found in a study with one of the largest series of patients (3). According to Du Geon Moon, the two main reasons for injection were a recommendation by acquaintances (49%) and to improve manliness (44%) (4). This complication can occur as early as 30 days to 30 years after injection. One of the highest incidences of this problem is reported in Thailand, a neighbouring country to Malaysia (3). The majority of our patients had their injection in that country and some of the processes were repeated over a period of months to years until the desired enlargement and shape were reached. Penile paraffinoma is usually associated with risky sexual behaviour in these patients (4). In contrast to our series, seven out of the eight patients were married and performed this procedure to improved sexual performance with their spouse.

Upon presentation, three of our patients who were injected only at the ventral side developed a focal mass formation on the penile shaft. These types of patients required a simple surgical procedure of excision of the mass and primary suturing of both lateral edges. Intra-operatively, the paraffinoma mass could be easily differentiated from the normal tissue and was completely excised with a sharp margin. Inadequate excision may result in re-growth of the mass. Surgical operation time was the shortest compared to the other techniques and achieved satisfactory outcomes.

Predominantly, patients had circumferential mass surrounding the penile shaft and extending to the suprapubic region. Meticulous en-bloc excision of the mass was performed, taking care not to breach the superficial fascia covering the corpus spongiosum and corpus cavernosum, which is the main reason for the longer duration of this technique. The next step was the reconstruction of the new penile skin. In our series, all patients underwent bilateral scrotal flap covering, a technique first described by Jeong et al. (5) in 1996. Scrotal skin is elastic and easily expands to cover the whole penile shaft. The uptake of this graft was always satisfactory, although it did not require any specific vessel to be preserved. Comparing the split skin graft, healing is faster and tactile sensibility is still intact. In our series, four patients underwent this technique in a single setting and one underwent it in two stages. There was no major difference in the outcome from both settings. In the single-stage, a square-shaped skin graft was sufficient, whereas in the dual-stage, the zigzag shape was a better option. Despite minor surgical site infection post-operatively, all patients recovered fully from this type of procedure.

None of our patients experienced erectile dysfunction post-operatively and could usually perform sexual intercourse 6 weeks–8 weeks after the procedure. According to Pungrasmi et al. (6), satisfaction scores of sexual activities significantly improved after adequate surgical procedure. Despite favourable outcome, this report has limited number of patients to make significant comment on the side effect and drawback from surgical correction of penile paraffinoma.

## Conclusion

Complications from penile augmentation using foreign body injection performed by non-medical personnel were common. In our experience, excision biopsy was adequate for focal mass and reconstructive surgery using bilateral scrotal flap was the best option for circumferential mass. Larger series and longer follow up are needed to determine the best surgical technique in treating this disease.

## Figures and Tables

**Table 1:** Summary of demography data, presentation and surgical procedure

No	Age	Race	Substance injected	Presentation	Examination finding	Procedure performed	Duration
1	47	Chinese	Unsure	Pain during sexual intercourse	Circumferential mass and ulcer	Dual stage procedure	1st 120 min 2nd 260 min
2	27	Malay	Paraffin	Persistent pain	Circumferential mass	Single stage procedure	225 min
3	27	Malay	Paraffin	Abnormal lump	Focal mass at ventral shaft	Simple excision biopsy	140 min
4	28	Malay	Silicone	Pain during sexual intercourse	Circumferential mass	Single stage procedure	245 min
5	25	Chinese	Unsure	Pain during sexual intercourse	Focal mass at ventral shaft	Simple excision biopsy	135 min
6	24	Indian	Unsure	Persistent pain	Circumferential mass	Single stage procedure	270 min
7	34	Malay	Paraffin	Abnormal lump	Circumferential mass with ulcer	Single stage procedure	345 min
8	72	Malay	Unsure	Abnormal lump	Focal mass at ventral shaft	Simple excision biopsy	160 min
